# Cell membrane patches transfer CAR molecules from a cellular depot to conventional T cells for constructing innovative fused-CAR-T cells without necessitating genetic modification

**DOI:** 10.1186/s40164-024-00545-z

**Published:** 2024-08-05

**Authors:** Jing Hu, Luyi Zhong, Yiqiu Wang, Shiyi Hu, Lijiaqi Zhang, Qingchang Tian

**Affiliations:** 1 School of Pharmacy, Hangzhou Normal University, Hangzhou, Zhejiang 311121 China; 2 Key Laboratory of Elemene Class Anti-Cancer Chinese Medicines, Engineering Laboratory of Development and Application of Traditional Chinese Medicines, Collaborative Innovation Center of Traditional Chinese Medicines of Zhejiang Province, Hangzhou, Zhejiang 311121 China

**Keywords:** CAR-T cell, Cell membrane patches, CAR molecule, T cell

## Abstract

Chimeric antigen receptor (CAR) serves as the foundational element of CAR-T cells. Exogenous CAR molecules can exert functional effects on allogeneic T cells, leading to their activation and subsequent functional alterations. Here we show a new method based on this biological principle: the transfer of CAR molecules from exogenous cells to the membrane of receptor T cells. This process facilitates receptor T cell to recognize target antigens and induces their activation. These patches imbued normal T cells with enhanced tumor targeting capabilities and activated their inherent killing functions. This method’s efficacy introduces an approach for constructing non-genetically manipulated CAR-T cells and holds potential for application to other immune cells.

**Graphical Abstract:**

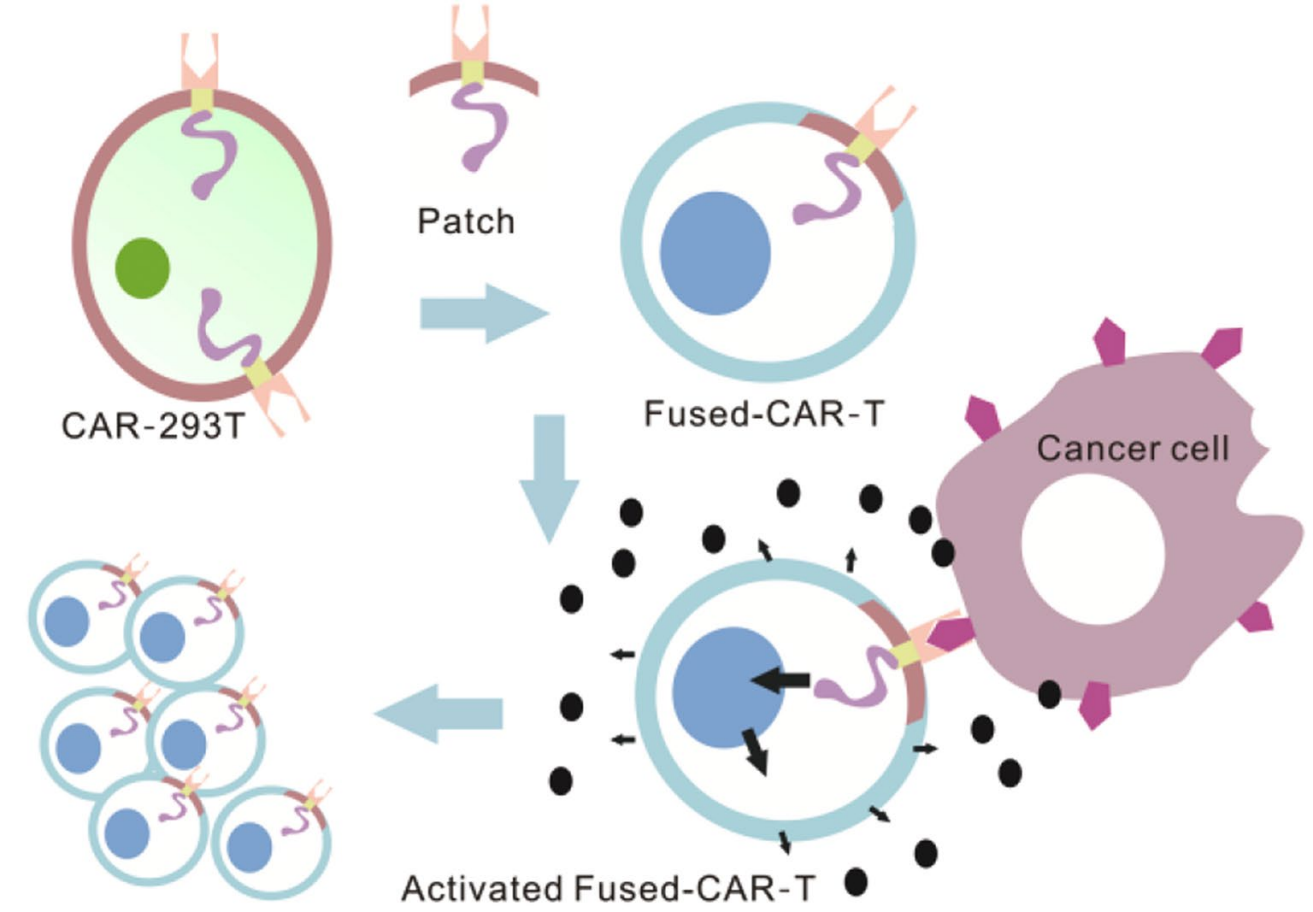

**Supplementary Information:**

The online version contains supplementary material available at 10.1186/s40164-024-00545-z.

## To the editor,

Chimeric antigen receptor (CAR) constitutes the core element of CAR-T cells, conferring T cells with an enhanced capacity to recognize tumor antigens [1, 2]. The fabrication of CAR-cells, such as CAR-T, predominantly relies on viral and non-viral genetic engineering techniques. A new safety notice about CAR-T cell therapy was reported [3]. With advancements in mRNA technology [4], modified mRNA encoding CAR to T lymphocytes was developed [5, 6], which produced transient, effective CAR-T cells in vivo to treat cardiac injury [6, 7]. However, targeted approaches are required to further enhance therapeutic effects.

As CAR is a fusion protein, it bridges the gap between antibody molecules and T-cell activation signaling molecules. This fusion facilitates the activation of downstream signals, thereby augmenting cell recognition and activation [2, 8]. Proteins can function in other cells while preserving their activity, and even nanosized plant-derived photosynthetic systems enable photosynthesis in animal cells [9]. Based on biological principles, exogenous CAR molecules can exert functional effects on allogeneic T-cells, leading to their activation and subsequent functional alterations.

This study provides proof of concept based on the biological principle of transferring CAR molecules from exogenous cells to the membrane of receptor T cells. (Fig. S1A). The efficacy of this method offers a novel strategy for constructing non-genetically manipulated CAR-T cells and shows promise for use with other types of immune cells.

**Fig. 1:**
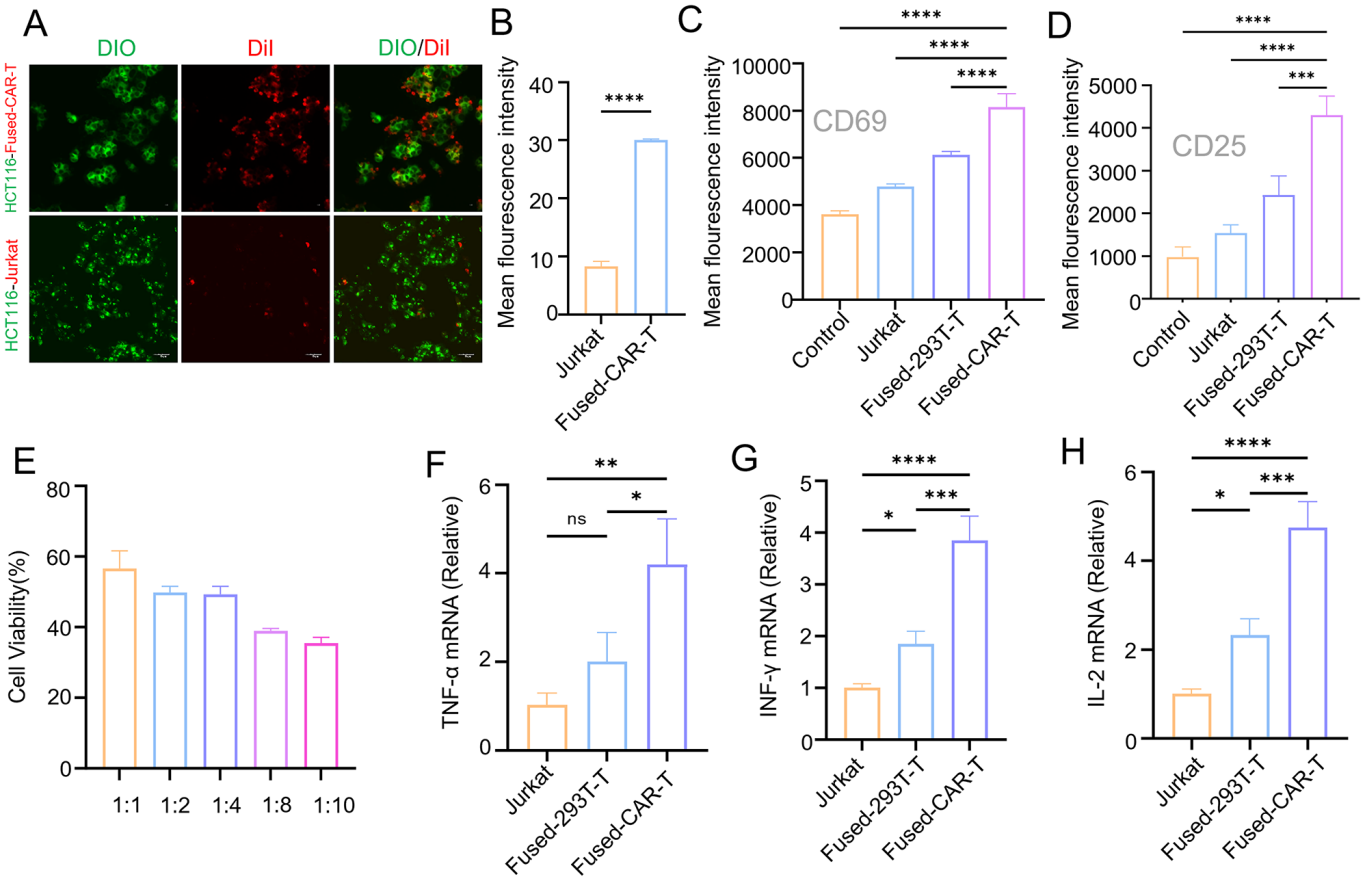
Fused-CAR-T cells were activated by tumor antigens. (**A**, **B**) The fluorescence signal of fused-CAR-T cells and HCT116 cells was monitored using confocal microscopy, (*n* = 3, mean ± SD). ****P < 0.0001. (**C**-**D**). Flow cytometry results revealed the expression of CD69 and CD25 protein on different cell lines stimulated by HCT116 cells, (*n* = 3, mean ± SD), ****P < 0.0001. (**E**) CCK8 assays were employed to measure the killing ability of fused-CAR-T cells against HCT116 cells at different E: T ratios, (*n* = 3, mean ± SD). (**F**-**H**) The gene expressions of IFN-γ, TNF-α, IL-2 in T cells were measured by PCR, (*n* = 3, mean ± SD), ns, no significant difference, *P < 0.05, ** *P* < 0.01, ***P < 0.001, ****P < 0.0001

CAR donor cells (specifically, CAR-293T cells) were engineered by introducing the scFv(anti-EpCAM)-41BB-CD3ζ expression gene into 293T cells, and CAR molecules were subsequently confirmed by flow cytometry and immunoblotting using a CD3ζ antibody (Figs. S1B-S1D). We fused these CAR-293T cell membrane patches with Jurkat cells at 37 °C using the fusion agent PEG1450. We initially assessed their ability of fused-CAR-T cells to target EpCAM-positive cells. Results indicated that the group of fused-CAR-T cells exhibited superior targeting ability, demonstrating a stronger capture rate of EpCAM-positive cells (Fig. 1A and B).

CAR-T cells can be activated by tumor antigens in the presence of exogenous CAR molecules. The findings revealed that, upon stimulation by HCT116 cells, a significant increase was observed in the surface proteins of fused-CAR-T cells, particularly in CD69 and CD25 molecules (Fig. 1C and D). This suggests that the fused-CAR-T cells exhibit a clear activation status following stimulation by the target cell HCT116 cells. As the E: T ratios (2:1, 4:1, 8:1, and 10:1) consistently increased, a corresponding gradual enhancement was found in the tumor-killing capability of fused-CAR-T cells, demonstrating a dose-dependent relationship (Fig. 1E). The aforementioned findings demonstrate that membrane patches derived from CAR-293T cells can transport CAR molecules to T cells, thereby facilitating tumor targeting and T cell activation.

Fused-CAR-T cells, stimulated by tumor antigens, release a significant quantity of cytokines to eliminate tumor cells. Among these, the cytokines IFN-γ and TNF-α are crucial indicators for assessing the antitumor efficacy of CAR-T cells. The expression of in vitro killing effect marker genes was examined (Fig. 1F and H). The findings revealed that the gene expression levels of IFN-γ, TNF-α, and IL-2 in fused-CAR-T cells exceeded those in the other groups.

**Fig. 2:**
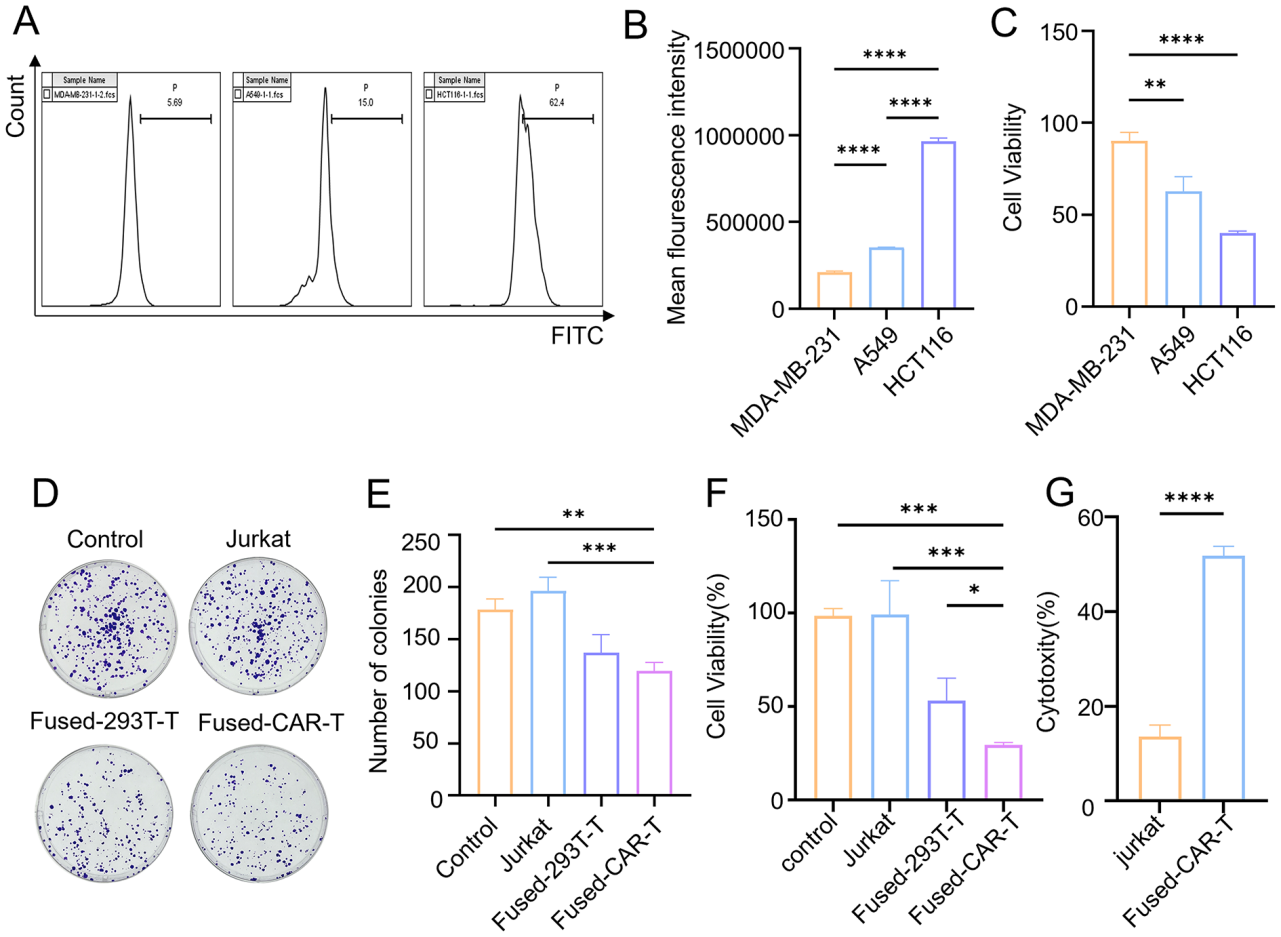
The in vitro tumor cell-killing capability of fused-CAR-T cells. (**A**-**B**) Flow cytometry was employed to detect the expression of EpCAM antigen on MDA-MB-231, A549, and HCT116 cell lines, (*n* = 3, mean ± SD), ****P < 0.0001. (**C**) CCK8 assays were utilized to measure the killing ability of fused-CAR-T cells against different tumor cells at E: T ratio of 10:1, (*n* = 3, mean ± SD), *P < 0.05, ** *P* < 0.01, ***P < 0.001, ****P < 0.0001. (**D**-**E**) The plate clone formation assays were deployed to measure the killing ability of fused-CAR-T cells against HCT116 cells, (*n* = 3, mean ± SD), ** *P* < 0.01, ***P < 0.001. (**F**-**G**) CCK8 and LDH release assays were used to measure the killing ability of different T cells against HCT116 cells at E: T ratio of 10:1, (*n* = 3, mean ± SD), *P < 0.05, ***P < 0.001, ****P < 0.0001

To confirm the in vitro tumor cell-killing capability of fused-CAR-T cells, the findings revealed a higher expression of EpCAM antigen in HCT116 cells than in A549 and MDA-MB-231 cells (Fig. 2A and B). At an E: T ratio of 10:1, the killing efficacy of fused-CAR-T cells against tumor cells was the highest in HCT116 cells, followed by A549 and MDA-MB-231 cells (Fig. 2C). Furthermore, the plate clone formation assay demonstrated that the number of HCT116 cell clones in the fused-CAR-T cell group was significantly lower than that in the Jurkat cell group (Fig. 2D and E). Based on the results from the CCK8 assays and lactate dehydrogenase (LDH) release assays, under identical E: T ratios, the killing effect of fused-CAR-T cells on HCT116 was more pronounced than other T cells (Fig. 2F and G). These findings underscore that exogenous CAR molecules confer enhanced targeting and cytotoxicity to conventional T cells.

The production and release of cytokines are the pathway for CAR molecules to exert their effects. The secretion of cytokines such as IL-2, TNF-α, and IFN-γ can be used to evaluate the activation status and function of CAR-T cells. The results showed a significant elevation in cytokine expression within fused-CAR-T cells following co-cultivation with HCT116 cells, herein referred to as co-fused-CAR-T (Figs. S2A-S2F), suggesting that the fused-CAR could enhance the activation state of CAR-T cells. These data imply that conventional T cells can achieve activation and express cellular functional genes when influenced by transferred CAR molecules.

To sum up, CAR-293T cell is a “cell depot” in this study, generating many CAR member patches. Fused-CAR-T cells were subsequently prepared using the member fusion technology. These patches imbued normal T cells with enhanced tumor targeting capabilities and activated their inherent killing functions. This approach facilitates the integration of exogenous CAR elements into normal T cells, expediting the construction of non-gene-modified CAR-T cells. Consequently, this study offers a novel perspective for developing universal CAR-T cells and other CAR variants without necessitating genetic modification.

### Electronic supplementary material

Below is the link to the electronic supplementary material.

Supplementary Material 1

## Data Availability

No datasets were generated or analysed during the current study.

## Abbreviations

CAR: Chimeric antigen receptor
IFN-γ: Interferonγ
TNF-α: Tumor necrosis factorα
IL-2: Interleukin 2
LDH: Lactate dehydrogenase
CCK8: Cell counting kit-8
E:T: Efficacy to target
