# Supplementary material for: Cell membrane patches transfer CAR molecules from a cellular depot to conventional T cells for constructing innovative fused-CAR-T cells without necessitating genetic modification
Source: Exp Hematol Oncol. 2024 Aug 5;13:75. doi: 10.1186/s40164-024-00545-z (PMC11302086; doi:10.1186/s40164-024-00545-z)
Supplement: Supplementary file 1 — Supplementary Material 1 [file 40164_2024_545_MOESM1_ESM.docx]

Cell membrane patches transfer CAR molecules from a cellular depot to conventional T cells for constructing innovative fused-CAR-T cells without necessitating genetic modification

Jing Hu^1,2^, Luyi Zhong^1^, Yiqiu Wang^1,2^, Shiyi Hu^1,2^, Lijiaqi Zhang^1,2^, Qingchang Tian^1,2*^

^1^ School of Pharmacy, Hangzhou Normal University, Hangzhou, Zhejiang 311121, China.

^2^ Key Laboratory of Elemene Class Anti-Cancer Chinese Medicines; Engineering Laboratory of Development and Application of Traditional Chinese Medicines; Collaborative Innovation Center of Traditional Chinese Medicines of Zhejiang Province, Hangzhou Normal University, Hangzhou, Zhejiang 311121, China.

*Correspondence:

Qingchang Tian

tianqc@hznu.edu.cn

**Methods**

Materials

All DNA primers utilized in the PCR process were synthesized and purified at Tsingke Biotechnology Co., Ltd. (Beijing, China). The calcein-AM/PI, Double Stain Kit, 3,3-dioctadecyloxacarbocyanine perchlorate (Dio), and 1,1′-Dioctadecyl-3,3,3′，3′-tetramethylindocarbocyanine perchlorate (Dil) were procured from Yeasen Biotechnology Co., Ltd. (Shanghai, China). Additionally, 4',6-diamidino-2-phenylindole (DAPI) was sourced from Solarbio (Beijing, China). The Cell Counting Kit-8 (CCK-8) was obtained from Dalian Meilun Biotechnology Corporation (Dalian, China), and the LDH release assay was procured from Beyotime Biotechnology Corporation (Suzhou, China). The Goat Anti-Rabbit IgG H&L (Alexa Fluor® 488) CD3-ζ antibody (6B10.2) was acquired from Santa Cruz (sc-1239, China). TB Green Premix Ex Taq II for PCR was supplied by Takara (Japan), and AxyPrep^TM^ Multisource Total RNA was purchased from AXYGEN (Santa Clara, CA, USA). The antibodies used for immunofluorescence were provided by Abcam (Cambridge Science Park, UK), whereas flow-related antibodies were obtained from BioLegend (San Diego, CA, USA). The ELISA kits (IL-2, IFN-γ, TNF-α) were acquired from MultiSciences Biotech Co., Ltd. (Hangzhou, China). All oligonucleotides were dissolved in sterile water and stored at -20 °C.

Cell culture

CAR-293T cells were engineered by the Vigen Biotechnology Corporation (Zhenjiang, China). The cell lines Jurkat, A549, MDA-MB-231, and HCT116 cell lines were procured from the Chinese Academy of Science Cell Bank for Type Culture Collection (Shanghai, China). CAR-293T cells were cultivated in Dulbecco's modified Eagle Medium (DMEM) supplemented with 10% fetal bovine serum (FBS, ExCell Bio) and 1% penicillin/streptomycin, under conditions of 5% CO_2_ at 37 °C. Similarly, Jurkat, A549, and MDA-MB-231 cells were cultured in RPMI-1640 medium supplemented with 10% FBS and 1% penicillin/streptomycin. HCT116 cells were maintained in McCoy's 5A medium supplemented with 10% FBS and 1% penicillin/streptomycin under identical conditions as described above.

CAR donor cells (specifically, CAR-293T cells) were engineered by introducing the scFv(anti-EpCAM)-41BB-CD3ζ expression gene into 293T cells. To generate CAR-293T cell membrane patches abundant with CAR "elements", we employed a homogenizer to disrupt CAR-293T cells. Subsequently, we collected the resultant cell membrane patches and determined their particle sizes using a nanoparticle size analyzer. The analysis revealed that the particle sizes of the obtained membrane patches ranged between 1000 and 1500 nm.

CAR-293T cells and 293T cells were harvested and labeled with the CD3ζ antibody (Alexa Fluor® 488). The fluorescence intensity was then measured using flow cytometry. Proteins from CAR-293T cells and member patches were separated and subjected to 10% SDS–PAGE. Subsequently, they were analyzed by a western immunoblot assay using the CD3ζ antibody.

Preparation of fused-CAR-T

CAR-293T cells were harvested and rinsed twice with PBS to eliminate cellular debris and the culture medium. Subsequently, these cells were suspended in saline and homogenized using a TH-Mini homogenizer within a sterile 1.5 mL EP tube. Following this, cell member patches were isolated via centrifugation before being reconstituted in saline. The resultant product was then subjected to a nanoparticle potential meter for particle size measurements.

Jurkat cells (6*10^6^) were combined with 600 μL of CAR-293T cell membrane patches (10 mg/mL). A 1 mL drop of a 50% PEG 1450 fusion agent was added in 1 min at 37 °C to initiate the fusion process. The membrane-fused T cells were allowed to stand for 1 min, after which 1 mL of pre-warmed 10% DMEM was added dropwise within 30 s. This was followed by adding of an additional 3 mL of the same medium for another 30 s. Subsequently, the remaining medium (11 mL) was introduced into the centrifuge tube within 1 min to conclude the fusion of PEG1450. The centrifuge tube was then placed in a centrifuge at 1000 rpm for 5 min. This procedure was repeated two or three times to eliminate any unfused cell membranes, given the cells become fragile post-fusion, and the steps of centrifugal washing and resuspension should be minimized. A complete medium was subsequently added to rehydrate the T cells. To investigate the potential cytotoxicity induced by the fusion agent, we assessed the viability of fused-CAR-T cells using live/dead double staining and CCK8 assays.

To assess the fusion efficiency, CAR-293T cell membrane patches were stained with Dil (red, 5 μM), while Jurkat cells were stained with Dio (green, 5 μM). The presence of membrane-fused T-cells was subsequently detected using laser scanning confocal microscopy and flow cytometry.

Unfused Jurkat cells exhibited only green fluorescence, whereas the successfully constructed fused T cells displayed red and green fluorescence (Figs. S1E-S1F). This evidence substantiates the successful construction of fused-CAR-T cells. Survival rate was analyzed using calcein-AM/PI assay. Importantly, this cell fusion procedure did not compromise the activity of T cells (Fig. S1G). These membrane-fused T cells were subsequently designated fused-CAR-T cells.

The ability of fused-CAR-T cells to target HCT116 cells

To assess the targeting capability of fused-CAR-T cells towards HCT116 cells, we stained fused-CAR-T cells with Dil (red, 5 μM) and Jurkat cells with Dio (green, 5 μM). Both the cell types were co-incubated for 24 h. Subsequently, the cells were washed 3-6 times with PBS. The fluorescence intensity was then measured using laser scanning confocal microscopy and flow cytometry.

Activation of fused-CAR T cells

CAR-T cells can be activated by tumor antigens in the presence of exogenous CAR molecules. The activation was marked by the upregulation of CD69 and CD25 whose expressions on fused-CAR-T cells were examined by co-culturing them with HCT116 cells at an effect-to-target (E:T) ratio of 10:1.

Following a 48-h incubation period with HCT116 tumor cell adherent culture, the medium was removed, and the cells were subsequently rinsed once with PBS solution. Subsequently, T cells (5 million cells per well, maintaining an effector-to-target ratio of 1:10) were introduced into each group and incubated for an additional 48 h.

The aforementioned T cells were collected washed t2-3 times with PBS. Subsequently, the Human TruStain FcXTM blocking reagent was added to each group and incubated at room temperature blocking for 10 min (cell counting was performed at a rate of 5 μL antibody per million cells). Following this, the samples were centrifuged at 4 °C and 1000 rpm for 2-3 cycles. Then, 200 μL FITC anti-human CD3 antibody was added to each group (cell counting was performed at a rate of 5 μL antibody per million cells), incubated in the dark on ice for 30 min, followed by centrifugation at 4°C and 1000 rpm for three cycles. Next, 200 μL APC anti-human CD69/CD25 antibody was added to each group (cell counting was performed at a rate of 5 μL antibody per million cells), incubated in the dark on ice for 30 min, followed by centrifugation at 4°C and 1000 rpm for three cycles. Finally, the cells were resuspended in 300 μL PBS, processed them using a machine, and data were collected using a flow cytometer for analysis. It was essential to collect at least 1x10^4^ cells per sample.

Cytotoxicity of fused-CAR-T cells

The cytotoxicity of fused-CAR-T cells to EpCAM-positive cells was detected using CCK8 and LDH release assays. Fused-CAR-T cells to EpCAM-positive cells were co-incubated for 48 h. Fused-CAR-T cells were washed away with PBS for 3–6 times, and cell activity was detected using CCK8, LDH release and plate clone formation assays. With the continuous increase in the E:T ratios (2:1, 4:1, 8:1, 10:1), the cytotoxicity of fused-CAR-T cells was also detected by CCK8 and LDH release assays.

Measurement of marker gene expression

This study examined the expression of cytokines in fused-CAR-T cells both pre and post coculture (co-fused-CAR-T) with HCT116 cells. PCR and ELISA analysis further confirmed a notable rise in the concentrations of IL-2, IFN-γ, TNF-α in supernatant of fused-CAR-T cells.

In this study, the fused-CAR-T cell gene expression was measured using qRT-PCR. RNA was isolated from the cells using the Multisource Total RNA kit and reverse-transcribed into cDNA using HiScript Ⅲ qRT SuperMix. The genes were quantified by qRT-PCR using specific primers (Table s1). The concentrations of IL-2, IFN-γ, TNF-α was confirmed by ELISA kit.

**Table s1.** The primers used for RT-PCR.

| Primer | Sequence（5’-3’） | Source |
| --- | --- | --- |
| GAPDH (Forward) | CAATGACCCCTTCATTGACC | Tsingke Biotechnology Co. |
| GAPDH (Reverse） | GACAAGCTTCCCGTTCTCAG | Tsingke Biotechnology Co. |
| TNF-α (Forward） | CAGGCGGTGCTTGTTC | Tsingke Biotechnology Co. |
| TNF-α (Reverse） | TAGGAGACGGCGATGC | Tsingke Biotechnology Co. |
| IFNγ (Forward） | CTGTTACTGCCAGGACCCAT | Tsingke Biotechnology Co. |
| IFNγ (Reverse） | TCTGTCACTCTCCTCTTTCCAA | Tsingke Biotechnology Co. |
| IL-2 (Forward） | AGACCCAGGGACTTAATCAG | Tsingke Biotechnology Co. |
| IL-2 (Reverse） | ACAATGGTTGCTGTCTCATC | Tsingke Biotechnology Co. |
| IL-10 (Forward） | GACTTTAAGGGTTACCTGGGTTG | Tsingke Biotechnology Co. |
| IL-10 (Reverse） | TCACATGCGCCTTGATGTCTG | Tsingke Biotechnology Co. |
| IL-6 (Forward） | ACTCACCTCTTCAGAACGAATTG | Tsingke Biotechnology Co. |
| IL-6 (Reverse） | CCATCTTTGGAAGGTTCAGGTTG | Tsingke Biotechnology Co. |
| IL-4 (Forward） | CCAACTGCTTCCCCCTCTG | Tsingke Biotechnology Co. |
| IL-4 (Reverse） | TCTGTTACGGTCAACTCGGTG | Tsingke Biotechnology Co. |

Statistical analysis

All statistical analyses were performed using the GraphPad Prism 9.0. All quantitative results are presented as mean ± standard deviation (SD) of at least three independent replicates. Statistical significance between groups was assessed using a one-way analysis of variance (ANOVA). The threshold for statistical significance was *P* < 0.05.


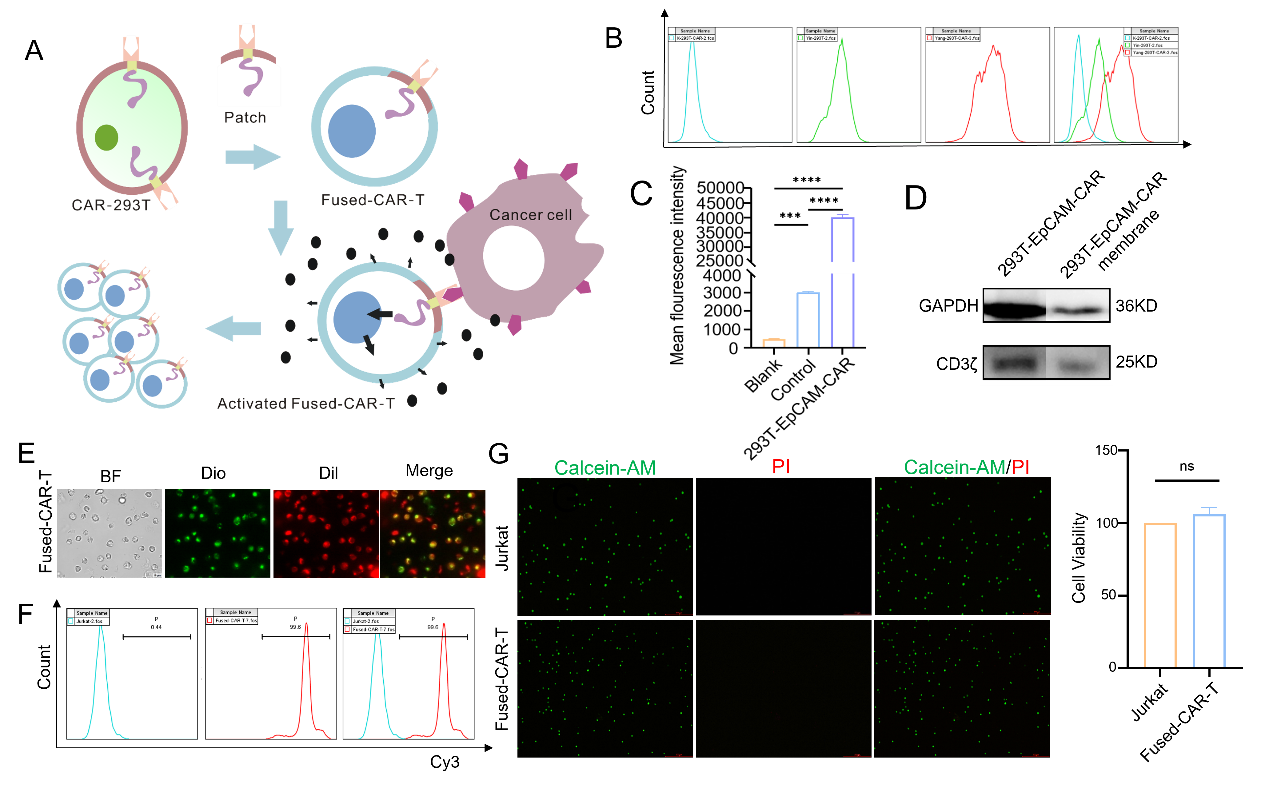


**Fig. S1** Cell membrane patches transfer CAR molecules for constructing fused-CAR T cells. (A) Illustration of fused-CAR T cells. These cells were constructed with membrane patches and activated for cytotoxicity. (B-C) CAR-293T cells were detected by flow cytometry using CD3ζ antibody (red) (n = 3, mean ± SD), ****P* < 0.001, *****P* < 0.0001. (D) CAR molecules in the CAR-293T membrane were assayed using immunoblotting. (E, F) The fluorescence signal of membrane-fused T cells was monitored by confocal microscopy and flow cytometry. (G) Survival rate was analyzed using calcein-AM/PI assay (n = 3, mean ± SD); ns, no significant difference (vs. Jurkat). The scale bar is 1000 μm.


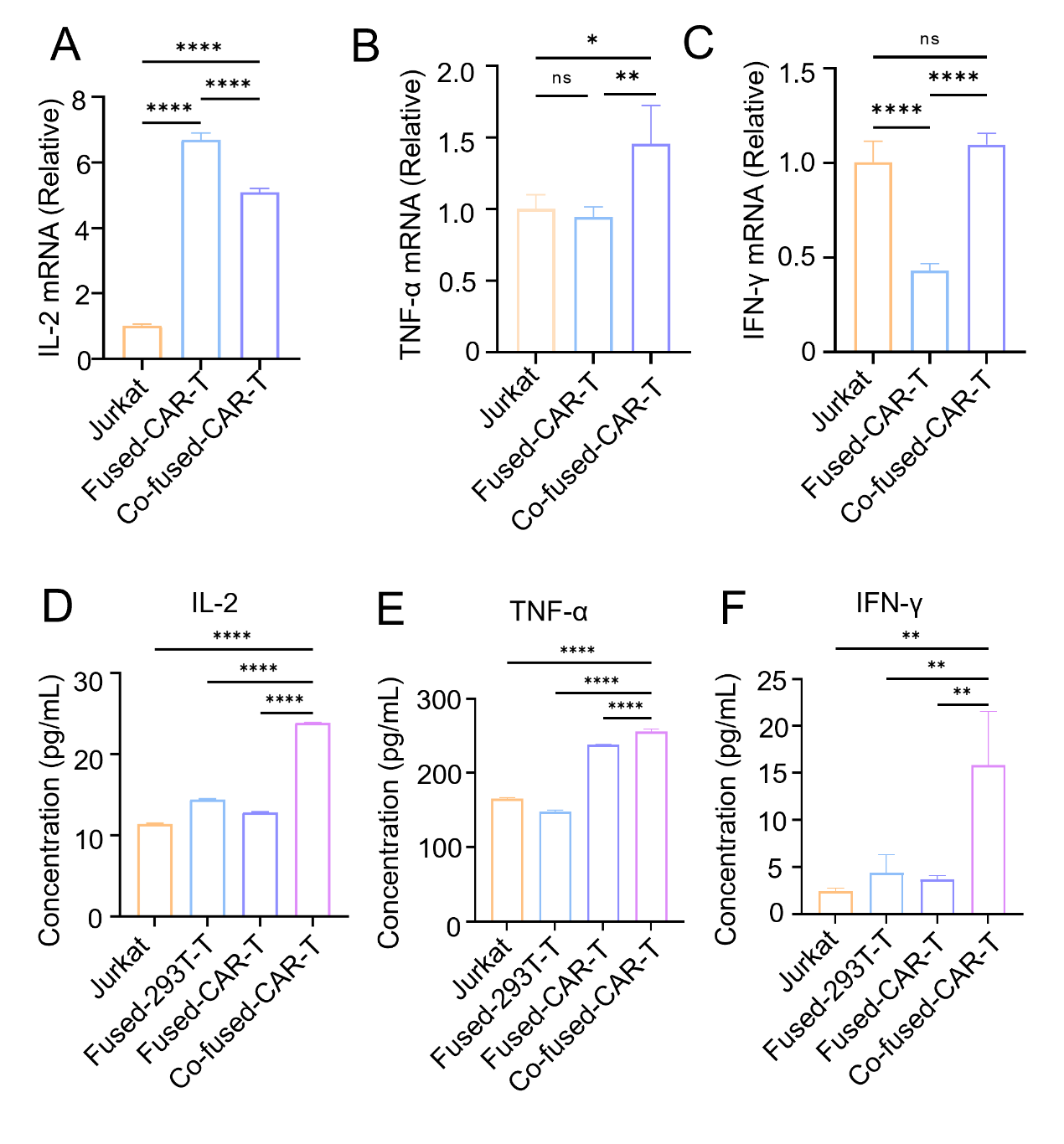


**Fig. S2** The expression of cytokines by co-fused-CAR-T cells. (A-C) The gene expressions of IFN-γ, TNF-α, IL-2 in T cells were measured by PCR, (n = 3, mean ± SD), ns, no significant difference, **P* < 0.05, ** *P* < 0.01, *****P* < 0.0001. (D-F) The concentrations of IL-2, IFN-γ, TNF-α in supernatant were measured by ELISA, (n = 3, mean ± SD), ns, no significant difference, ** *P* < 0.01, *****P* < 0.0001.
